# Improved performance of a barrier-discharge plasma jet biased by a direct-current voltage

**DOI:** 10.1038/srep35653

**Published:** 2016-10-19

**Authors:** Xuechen Li, Yaru Li, Panpan Zhang, Pengying Jia, Lifang Dong

**Affiliations:** 1 College of Physics Science & Technology, Hebei University, Baoding 071002, China; 2 Key Laboratory of Photo-Electronics Information Materials of Hebei Province, Baoding 071002, China

## Abstract

One of the challenges that plasma research encounters is how to generate a large-scale plasma plume at atmospheric pressure. Through utilizing a third electrode biased by a direct-current voltage, a longer plasma plume is generated by a plasma jet in dielectric barrier discharge configurations. Results indicate that the plume length increases until it reaches the third electrode with increasing the bias voltage. By fast photography, it is found that the plume consists of two types of streamers under the influence of the bias voltage, which develops from a guided streamer to a branching one with leaving the tube opening. The transition from the guided streamer to the branching one can be attributed to the electric field and the air/argon fraction.

Atmospheric pressure plasma jet (APPJ) has attracted a considerable interest recently due to a variety of reactive species in it^1^,^2^, such as meta-stable particle, OH radical and ozone. The abundance of reactive species stimulates extensive application potentials for biomedicine^3^,^4^,^5^, material modification and nanoparticle formation^6^,^7^.

Various configurations of APPJ have been investigated, such as dielectric-barrier discharge (DBD)^8^,^9^, hollow cathode discharge^10^,^11^, and corona discharge^12^. Usually, a plasma plume is generated in the gas channel of an APPJ, which represents the medium for the generation and propagation of the ionization wave. From the view point of discharge dynamics, most of the plasma plumes are composed of fast-moving plasma bullets (guided streamers)^13^,^14^, which propagate along a predetermined path (the gas flow direction)^15^. Moreover, the propagation track of the guided streamer can be straight-line^16^, snakelike or helical^17^,^18^. An electric field perpendicular to the gas flow has also been used to change the propagation direction of the guided streamer^19^. Besides the guided streamer, the other propagation behavior of the plasma plume is branching streamer^20^, which is similar to the large-gap discharge in a non-uniform field^21^. Hofmann *et al*. have pointed out that the plume in APPJ is stable branching streamers in the mixture of oxygen and helium, while it is random branching streamers in argon^20^. For a single-needle jet excited by a pulse voltage, Xiong *et al*. have found that the argon plume firstly behaves as the guided streamer, then as the non-reproducible branching streamer during its propagation process^22^.

The plume length is a noticeable parameter for APPJ^23^,^24^,^25^,^26^. Xiong *et al*. have found that the amplitude and pulse width of the voltage exert significantly stronger effects on the plume length than other parameters^23^. The plume length has also been investigated in various working gases (helium, argon, nitrogen, etc)^25^,^26^. A helium plasma plume has been reported by Lu *et al*. with length up to 11 cm in ambient air^27^. Compared with the helium jet, the plasma plume length is not satisfactory with argon used as the working gas^25^. Therefore, one of the challenges that currently face plasma research is how to generate a longer plasma plume in inexpensive gases^28^.

In this paper, through utilizing a third electrode biased by a direct-current (DC) voltage, a longer plasma plume is generated downstream of an argon DBD plasma jet. It is found that the plasma plume behaves as the branching streamer besides the guided streamer.

## Results

**Figure 1:**
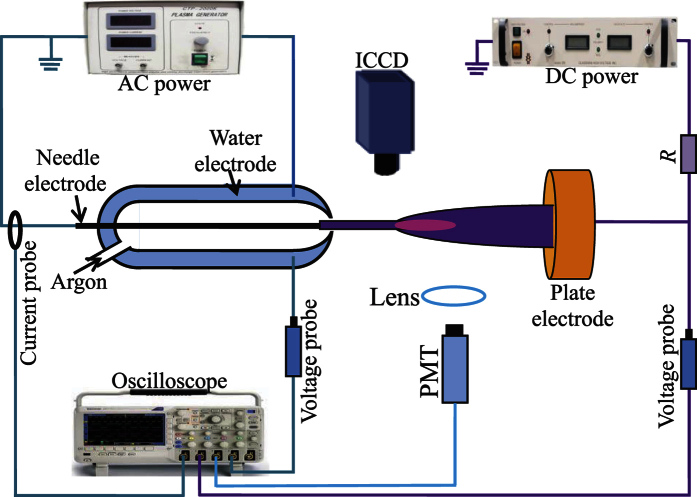
Schematic diagram of the experimental setup.

**Figure 2:**
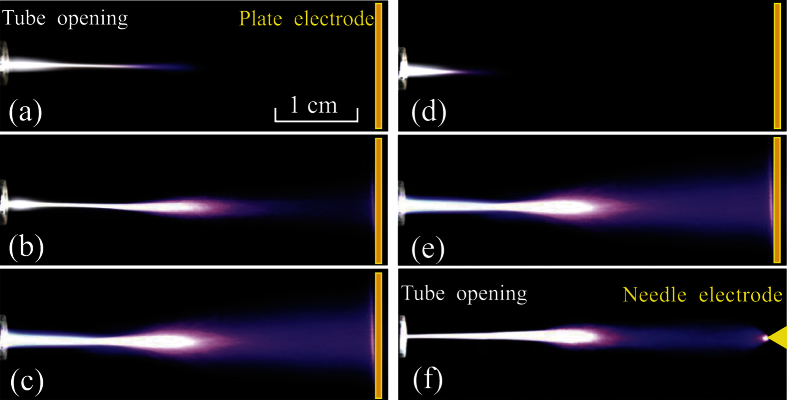
Discharge images of the plasma plumes between the coaxial DBD jet and the plate electrode under different *V*_*b*_: (**a**) 0 kV, (**b**) 10 kV, (**c**) 15 kV. (**d**,**e**) correspond to (**a**,**c**), respectively, except that a single-electrode jet is used. (**f**) is the same conditions with (**c**) except that the plate electrode is replaced by the tungsten needle. The exposure time is 0.1 s.

Figure 1 presents the schematic diagram of the experimental setup. When the DBD peak voltage (*V*_*D*_) increases to 4.5 kV and the DC biased voltage (*V*_*b*_ denotes its absolute value) is zero, the DBD jet generates a straight-line-shaped plume in the vicinity of the tube opening, as shown in Fig. 2(a). With increasing *V*_*b*_, it is found that the plume length increases until a cup-shaped plume bridges the tube opening and the plate electrode, as shown in Fig. 2(b,c). Compared with Fig. 2(b), the cup-shaped plume in Fig. 2(c) becomes more luminous and extends more on the plate electrode. Usually, an APPJ generates plasma in free space rather than inside of a gap between electrodes. Strictly speaking, the plasma generated by the third electrode is not a plasma jet any more since it is generated within a gap. However, this definition about plasma jet can be extended, for example, the plasma generated between a needle electrode and a plate electrode is also categorized as a plasma jet^1^,^29^. Therefore, we still call it a plasma jet though utilizing the third electrode to improve the performance of the plasma jet in coaxial DBD configurations.

The effect of the electrode configuration is investigated on the discharge behavior. Firstly, the water electrode is removed and the tungsten electrode is connected with the output of the AC power supply, which constitutes a single-electrode jet. From Fig. 2(d), it is found that the single-electrode jet with zero *V*_*b*_ generates a straight-line-shaped plume in the vicinity of the tube opening, whose length is shorter than that of the coaxial DBD jet in Fig. 2(a). With increasing *V*_*b*_ to 15 kV (Fig. 2(e)), the plume length also increases and bridges the tube opening and the plate electrode. Therefore, the biased third electrode can also improve the performance of the single-electrode jet. Consequently, whether the plasma jet is in coaxial DBD configurations or single-electrode one, a longer plasma plume will be generated with the third electrode biased by a DC voltage. Secondly, a tungsten needle (same with the tungsten electrode used in the DBD jet) is used to replace the plate as the third electrode. Compared with the plume in Fig. 2(c), the plume influenced by the needle electrode has a smaller cross section, as shown in Fig. 2(f).

**Figure 3:**
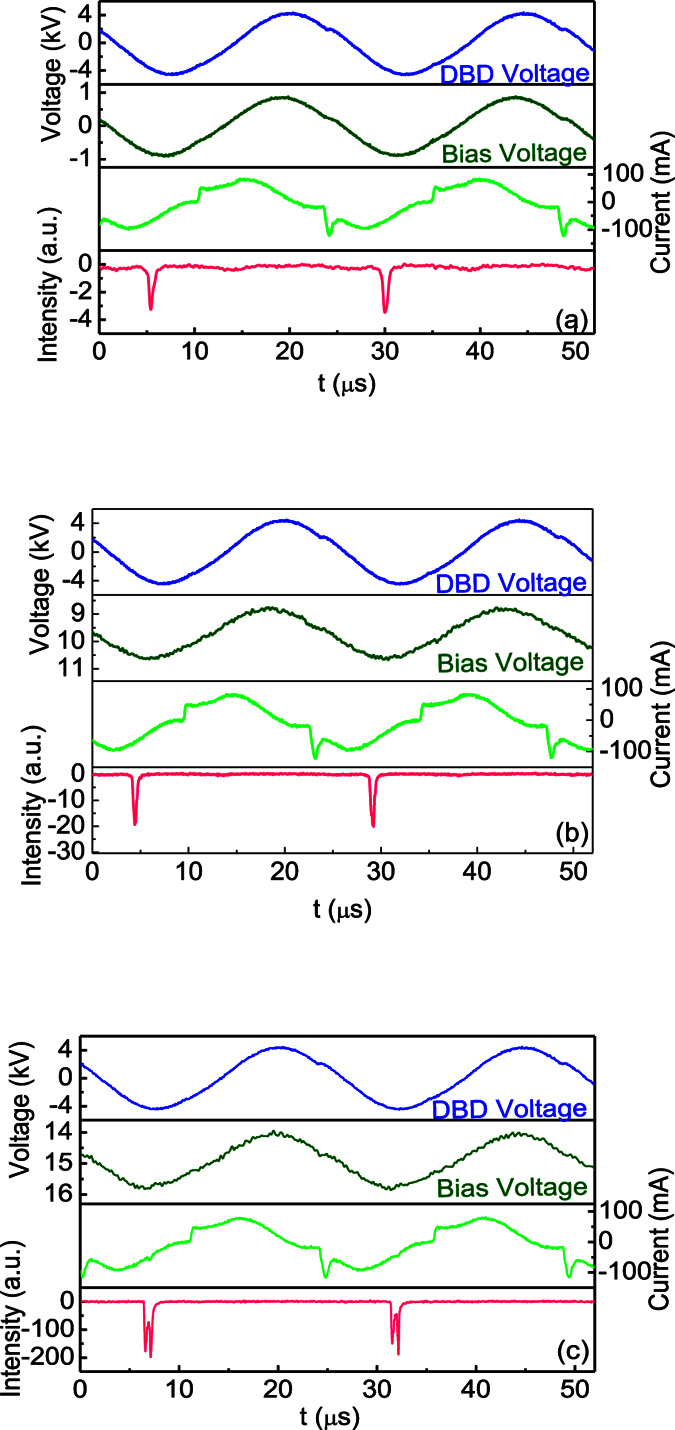
Waveforms of the DBD voltage, the bias voltage, the current and the light emission under different *V*_*b*_: (**a**) 0 kV, (**b**) 10 kV, (**c**) 15 kV.

Figure 3 shows the waveforms of the DBD voltage, the bias voltage, the current and the light emission. Despite a DC power supply is used, the bias voltage between the plate electrode and the tungsten one oscillates under the influence of the DBD voltage. The frequency of bias voltage is equal to that of the DBD voltage. The plume discharge is an ordinary DBD because of zero *V*_*b*_, therefore, the current in Fig. 3(a) is composed of discharge current and displacement one^30^,^31^. The current waveforms keep almost unchanged in both shape and amplitude with increasing *V*_*b*_. Hence, the light signal emitted from the discharge region is investigated between the opening tube and the plate. It is found that the discharge pulse only appears in the negative half cycle of the voltage under different *V*_*b*_. Therefore, the discharge is generated when the tungsten electrode is the instantaneous anode. Moreover, it is found that the light emission intensity increases with increasing *V*_*b*_. In addition, the pulse number doubles when *V*_b_ reaches 15 kV.

**Figure 4:**
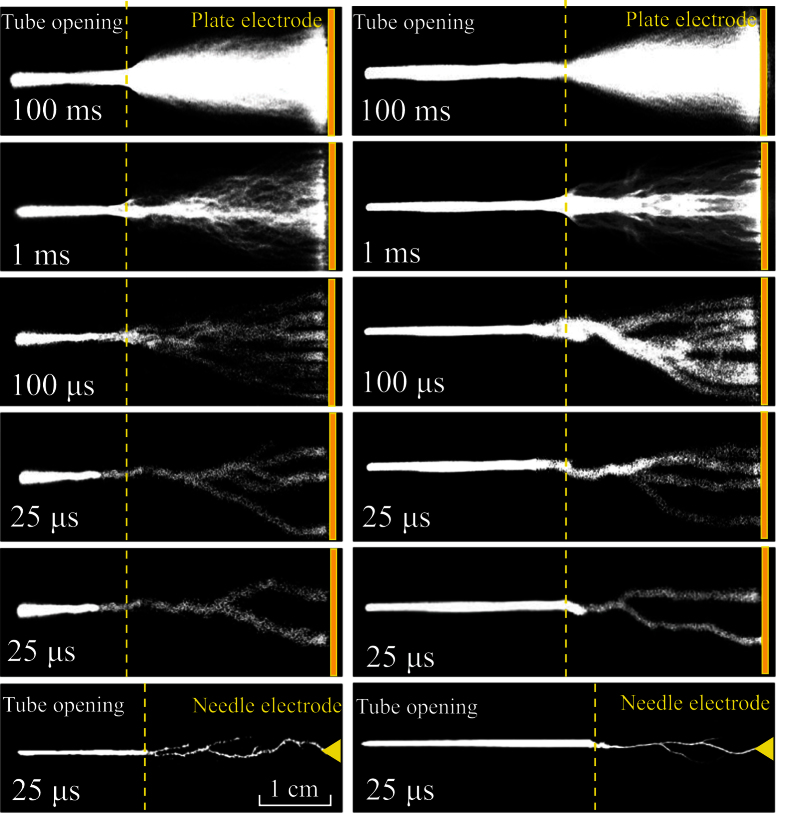
Discharge images with argon (the left) or helium (the right) used as the working gas taken by the ICCD under different exposure times. The *V*_b_ is 14 kV.

Figure 4 presents the discharge images in argon (the left) and helium (the right) under different exposure times. The exposure time is labeled in every image of Fig. 4. It is found that, with decreasing exposure time, all of the discharge tracks remain a straight line on the left side of the dashed line. However, discrete branches appear on its right side. It should be emphasized that the images with an exposure time of 25 μs are captured at different moments. Hence, the discharge structure is reproducible for the straight-line part, while it is of high non-reproducible for the branching part. Compared with the plate electrode, the plume with the needle used as the third electrode has a longer straight-line part and a branching part with a smaller cross section. To avoid the transition into a spark discharge, a larger gap width (5.5 cm) is used for the helium jet.

**Figure 5:**
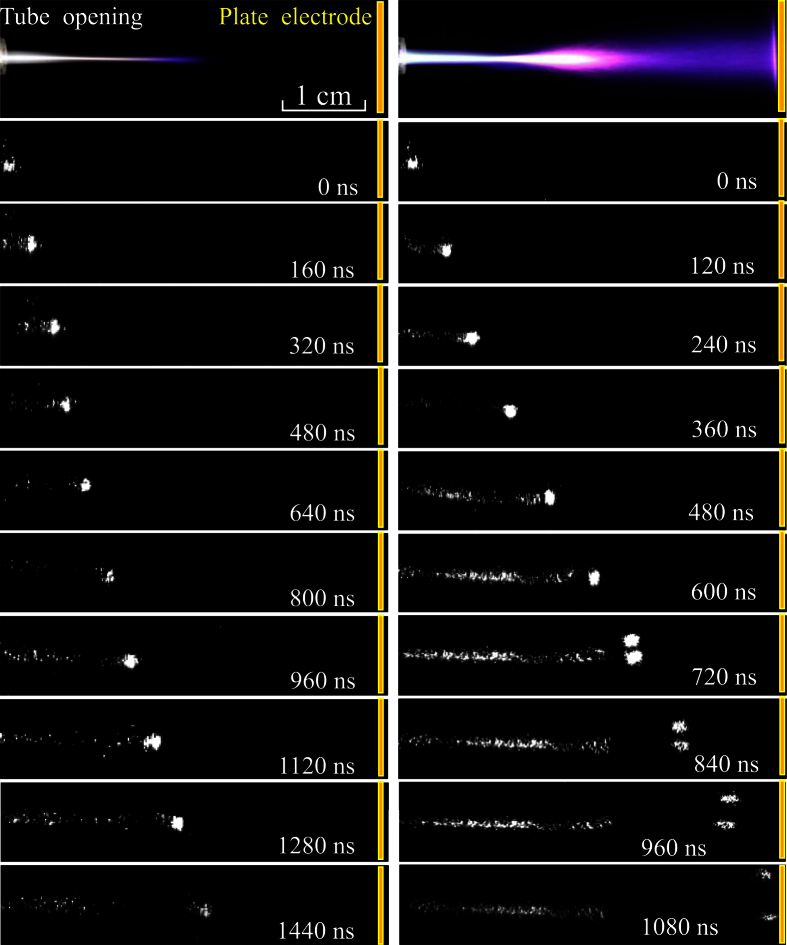
Temporal evolution of the plasma plumes under different *V*_*b*_: 0 kV (the left), and 14 kV (the right). Single shot for each image with an exposure time of 10 ns.

The effect of the bias voltage on the discharge dynamics is further investigated with high-speed optical imaging with an exposure time of 10 ns. In Fig. 5, the temporal evolution of the plasma plumes are presented with *V*_*b*_ of 0 kV (the left) and 14 kV (the right), respectively. It is selected as the reference time 0 ns for the moment that the light emission just appears at the tube opening, which corresponds to the beginning of the discharge pulse. Obviously, the straight-line plume with zero *V*_*b*_ is composed of a fast-moving bullet (guided streamer) for every discharge cycle^13^,^14^. The bullet firstly appears near the tube opening at 0 ns, then propagates along the gas flow. At last, the bullet fades away at 1440 ns, about 2.5 cm away from the tube opening. For the discharge with 14 kV *V*_*b*_, a plasma bullet appears in the vicinity of the tube opening and propagates along a straight line from 0 ns to 600 ns, which is similar to that with zero *V*_*b*_ (the left). Later, the guided streamer transits into the branching one. The streamer splits into two heads at 720 ns. After that, the two streamer heads simultaneously propagate toward the plate electrode until they reach and extinguish there at 1080 ns. It is noteworthy that the branching streamer head appears stochastically in its number and position perpendicular to the gas flow. Consequently, the plume with bias voltage develops from the straight-line guided streamer to the branching one with leaving the tube opening. Compared with the streamer with zero *V*_*b*_, the propagation distance is farther and the propagation time is shorter for the streamer with 14 kV *V*_*b*_. Therefore, the averaged streamer velocity with 14 kV *V*_*b*_ (about 3.8 × 10^4 ^m/s) is higher than that with zero *V*_*b*_ (about 1.5 × 10^4 ^m/s).

**Figure 6:**
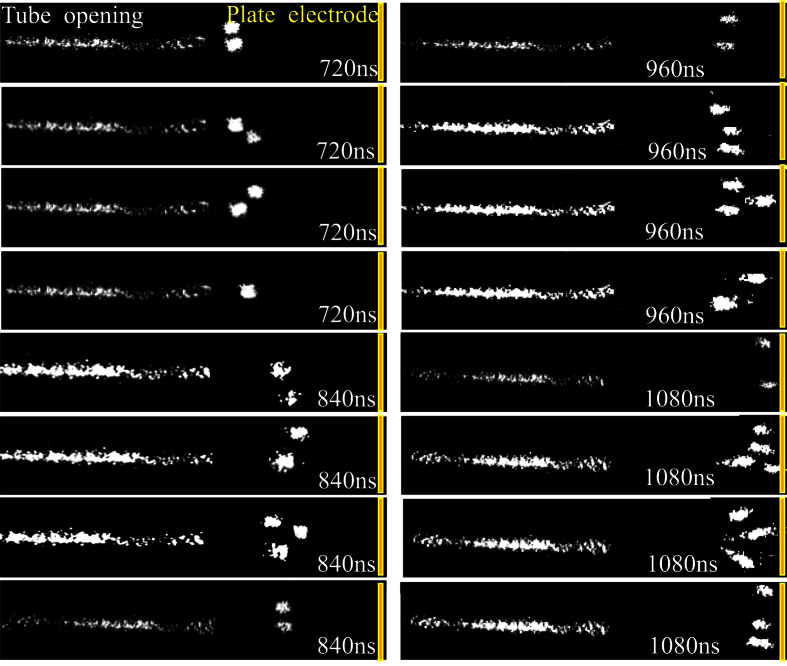
Four typical images of the propagating streamer for each time delay of the branching streamer stage, which corresponds to the right part of Fig. 5.

Due to the non-reproducibility of the propagating streamer, four typical images are shown in Fig. 6 for each time delay of the branching streamer stage. They are fairly random for both the number and the position of the branching streamer heads. Therefore, the spatial distribution probability of the streamer heads on the plate electrode is investigated statistically. It is found that the probability of the head is maximal at the center of the plate electrode, and gradually decreases along the radial direction of the plate electrode.

## Discussion

For the discharges with or without *V*_*b*_, the tungsten electrode of the coaxial DBD jet is the instantaneous anode. Therefore, the guided streamer is directed from the anode to the cathode, which is often called a cathode-directed streamer (or positive streamer)^16^,^32^. The propagation behavior of the positive streamer has been explained based on photo-ionization^29^,^32^. The streamer undergoes a scenario from launching, propagation to ending^33^, whose velocity firstly increases to a maximal value, remains almost constant, then decreases during its propagation process^16^,^33^. Moreover, the streamer velocity increases with increasing the electric field^34^. Obviously, the streamer does not extinguish until the net electric field decreases to a certain extent. Consequently, the streamer can only propagate a certain distance without *V*_*b*_. The electric field can be enhanced in front of the streamer head by *V*_*b*_. Therefore, the streamer can propagate a farther distance in a higher averaged velocity under the influence of the bias voltage.

Compared with the plate electrode, the electric field along the longitudinal direction is higher in the center for the needle used as the third electrode. Moreover, it decreases more abruptly with increasing distance from the center. Therefore, the higher longitudinal electric field in the center is beneficial for the propagation of the guided streamer. The generation of the branching streamer can be attributed to the higher longitudinal electric field in the region away from the center.

**Figure 7:**
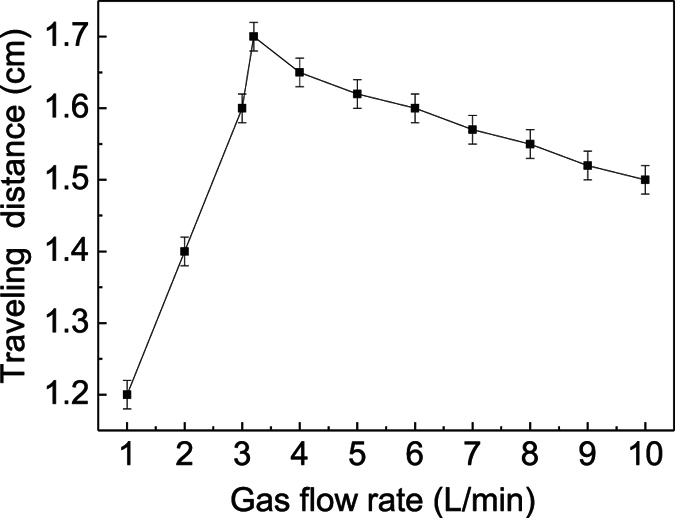
The traveling distance of the guided streamer as a function of the gas flow rate with *V*_*b*_ of 14 kV.

Xiong *et al*. have pointed out that the branching streamer propagation is related with the diffusion of the ambient air into the argon stream^22^. Therefore, the effect of the air/argon fraction on the streamer behavior is further investigated. From Fig. 7, it is found that the traveling distance of the guided streamer firstly increases, then decreases with increasing the flow rate. It reaches its maximal value at 3.2 L/min. After calculating the Reynolds number, it is a laminar flow with the argon flow rate less than 3.2 L/min, and a turbulent flow with a higher flow rate. In the laminar flow mode, the air/argon fraction decreases with increasing the flow rate^35^. In the turbulent mode, it increases with increasing the flow rate. Apparently, the air/argon fraction reaches its minimum value at 3.2 L/min. Therefore, traveling distance of the guided streamer increases with decreasing the air/argon fraction. It can be concluded that the plasma plume behaves more likely as the guided streamer under a lower air/argon fraction, while it tends to be the branching streamer under a higher fraction.

Consequently, the transition from the straight-line guided streamer to the branching one can be attributed to the electric field and the air/argon fraction.

## Methods

A schematic diagram of the experimental setup is shown in Fig. 1. A tungsten electrode (14 cm in length and 1 mm in diameter) has a sharp end with a tip radius of about 200 μm, which is coaxially wrapped by a double-layer tube (high borosilicate glass, 1 mm in thickness for each layer). The cavity is filled with tap water (PH value: about 6.5) for the double-layer tube (6 mm and 18 mm in inner diameter, respectively). The water is not flowing and keeps at a temperature lower than 35 °C through cooling by a water tank above the water electrode. Obviously, the water electrode and the tungsten one constitute the DBD jet in coaxial configurations, which is similar to the DBD configurations in our previous experiment^36^. By adjusting the peak value of the DBD voltage, the plasma jet operates in the linear-field mode. Argon or helium (99.999% purity) with a flow rate of 3.2 L/min is fed into the inner tube and flushes out through its conical opening (2.5 mm in diameter) into ambient air. Argon is used as the working gas if no special explanation is given. The distance is 13 mm from the tip of the grounded tungsten electrode to the conical tube opening. The water electrode is connected with 4.5 kV peak voltage generated by an alternating current (AC) power supply (Suman CTP-2000 K) with 40 kHz. A copper plate used as a third electrode is fixed at 4.5 cm away from the tube opening, which is negatively biased by a DC power supply (Glassman EK15R40) (amplitude up to 15 kV) through a ballast resistor (*R* = 500 kΩ) to prevent the undesired spark discharge. The DBD voltage and the bias voltage are detected by two high voltage probes (Tektronix P6015A). The current through the discharge circuit can be directly measured by a current probe (Tektronix TCPA300). Light emitted from the discharge is focused by a lens into a photomultiplier tube (PMT) (ET 9085SB). They are monitored simultaneously with an oscilloscope (Tektronix DPO4104). A digital camera (Canon EOS 7D) and an intensified charge-coupled device (ICCD) (Andor DH334) are used to capture the discharge images.

## Additional Information

**How to cite this article**: Li, X. *et al*. Improved performance of a barrier-discharge plasma jet biased by a direct-current voltage. *Sci. Rep.* **6**, 35653; doi: 10.1038/srep35653 (2016).
